# Supplementary material for: Role of Radiation Therapy in Mortality among Adolescents and Young Adults with Lymphoma: Differences According to Cause of Death
Source: Cancers (Basel). 2022 Oct 16;14(20):5067. doi: 10.3390/cancers14205067 (PMC9599966; doi:10.3390/cancers14205067)
Supplement: Supplementary file 1 [file cancers-14-05067-s001.zip › Table S1.pdf]

Table S1. Standardized mortality ratios of SMNs-related mortality among AYA patients according to baseline characteristics.

Characteristic	Radiation	No Radiation
	SMR(95% CI)	SMR(95% CI)
**Overall**	3.59*(2.84-4.48)	5.31*(4.54-6.18)
**Age, y**		
15-24	7.35*(4.2-11.94)	8.76*(5.49-13.27)
25-39	3.17*(2.43-4.07)	5.02*(4.24-5.9)
**Sex**		
Male	4.2*(3.07-5.62)	5.45*(4.41-6.67)
Female	3*(2.06-4.21)	5.14*(4.04-6.45)
**Race**		
White	3.47*(2.68-4.44)	5.39*(4.53-6.37)
Black	4.53*(2.17-8.34)	4*(2.41-6.25)
Other	3.66(1-9.36)	8.51*(4.25-15.22)
**Latency periods, m**		
0-11	8.81*(1.82-25.76)	11.61*(4.67-23.93)
12-59	5.48*(3.19-8.77)	13.26*(10.26-16.87)
60-119	3.89*(2.38-6.01)	5.46*(3.9-7.43)
120+	2.89*(2.05-3.97)	2.96*(2.24-3.85)
**Era of diagnosis, year**		
1992-2001	3.16*(2.36-4.15)	4.7*(3.87-5.65)
2002-2016	4.91*(3.21-7.2)	7.21*(5.44-9.36)
**Ann Arbor stage**		
I/II	2.65*(1.93-3.56)	3.68*(2.76-4.8)
III/IV	5.84*(3.85-8.5)	4.36*(3.38-5.52)
**lymphoma subtype**		
HL	3.25*(2.35-4.38)	3.74*(2.83-4.85)
DLBCL	3.1*(1.89-4.78)	3.02*(2-4.36)
BL	15.05*(3.1-43.97)	9.02*(3.89-17.77)
FL	3.28(0.89-8.4)	4.72*(2.84-7.38)
MZL	0(0-27.98)	0(0-6.78)
MCL	0(0-159.03)	9.28(0.23-51.71)
CLL/SLL	74.61*(30-153.74)	55.06*(41.25-72.02)
PTCL	2.68(0.07-14.95)	3.84(0.79-11.23)

*P<0.05

Abbreviations: AYA, adolescent and young adult; SMR, standardized mortality ratio; CI, confidence interval; HL, Hodgkin lymphoma; DLBCL, diffuse large B-cell lymphoma; MCL, mantle cell lymphoma; BL, Burkitt's lymphoma; MZL, marginal zone lymphoma; CLL/SLL, chronic lymphocytic leukemia/small lymphocytic lymphoma; PTCL, peripheral T-cell lymphoma; FL, follicular lymphoma; SMNs, second malignant neoplasms.
