# Supplementary material for: Role of Radiation Therapy in Mortality among Adolescents and Young Adults with Lymphoma: Differences According to Cause of Death
Source: Cancers (Basel). 2022 Oct 16;14(20):5067. doi: 10.3390/cancers14205067 (PMC9599966; doi:10.3390/cancers14205067)
Supplement: Supplementary file 1 [file cancers-14-05067-s001.zip › Table S2.pdf]

Table S2. Standardized mortality ratios of noncancer diseases-related mortality among AYA patients according to baseline characteristics.

Characteristic	Radiation	No Radiation
	SMR(95% CI)	SMR(95% CI)
**Overall**	2.45*(2.15-2.77)	4.56*(4.23-4.91)
**Age, y**		
15-24	2.15*(1.59-2.85)	3.03*(2.43-3.74)
25-39	2.53*(2.19-2.91)	4.91*(4.53-5.31)
**Sex**		
Male	2.53*(2.18-2.93)	5.04*(4.63-5.47)
Female	2.26*(1.77-2.84)	3.24*(2.71-3.84)
**Race**		
White	2.32*(2.01-2.67)	4.41*(4.05-4.81)
Black	2.97*(2.11-4.06)	4.74*(3.98-5.6)
Other	3.37*(1.89-5.56)	7.1*(4.94-9.87)
**Latency periods, m**		
0-11	17.44*(13.17-22.65)	28.43*(24.29-33.06)
12-59	2.67*(2.06-3.4)	6.81*(6.02-7.66)
60-119	1.35(0.96-1.85)	2.68*(2.2-3.22)
120+	1.97*(1.59-2.42)	2.25*(1.9-2.64)
**Era of diagnosis, year**		
1992-2001	2.71*(2.34-3.14)	4.76*(4.35-5.21)
2002-2016	1.93*(1.49-2.45)	4.18*(3.65-4.77)
**Ann Arbor stage**		
I/II	1.78*(1.5-2.11)	3.27*(2.86-3.72)
III/IV	4.47*(3.65-5.41)	5.67*(5.15-6.23)
**lymphoma subtype**		
HL	1.7*(1.4-2.05)	2.88*(2.52-3.28)
DLBCL	3.36*(2.72-4.12)	7.99*(7.16-8.89)
BL	15.21*(9.02-24.05)	9.71*(7.41-12.5)
FL	3*(1.6-5.13)	1.95*(1.31-2.81)
MZL	5.29*(1.09-15.46)	4.47*(2.14-8.22)
MCL	0(0-33.64)	5.85*(1.21-17.08)
CLL/SLL	10.25*(2.79-26.26)	4.33*(2.57-6.85)
PTCL	3.54*(1.3-7.7)	3.77*(2.19-6.03)

*P<0.05

Abbreviations: AYA, adolescent and young adult; SMR, standardized mortality ratio; CI, confidence interval; HL, Hodgkin lymphoma; DLBCL, diffuse large B-cell lymphoma; MCL, mantle cell lymphoma; BL, Burkitt's lymphoma; MZL, marginal zone lymphoma; CLL/SLL, chronic lymphocytic leukemia/small lymphocytic lymphoma; PTCL, peripheral T-cell lymphoma; FL, follicular lymphoma.
