# Supplementary material for: Role of Radiation Therapy in Mortality among Adolescents and Young Adults with Lymphoma: Differences According to Cause of Death
Source: Cancers (Basel). 2022 Oct 16;14(20):5067. doi: 10.3390/cancers14205067 (PMC9599966; doi:10.3390/cancers14205067)
Supplement: Supplementary file 1 [file cancers-14-05067-s001.zip › Table S3.pdf]

Table S3. Standardized mortality ratios of infection-related mortality among AYA patients according to baseline characteristics.

Characteristic	Radiation	No Radiation
	SMR(95% CI)	SMR(95% CI)
**Overall**	14.67*(12.01-17.74)	33.04*(29.85-36.48)
**Age, y**		
15-24	9.78*(4.47-18.57)	21.2*(13.85-31.06)
25-39	15.38*(12.47-18.76)	34.41*(30.97-38.12)
**Sex**		
Male	19.56*(15.81-23.94)	41.19*(36.99-45.73)
Female	4.95*(2.56-8.65)	12.27*(8.81-16.65)
**Race**		
White	15.37*(12.19-19.14)	37.43*(33.22-42.02)
Black	11.88*(7.36-18.17)	22.32*(17.88-27.53)
Other	19.41*(6.3-45.29)	60.47*(35.84-95.57)
**Latency periods, m**		
0-11	174.66*(128.78-231.58)	255.75*(215.42-301.44)
12-59	19.64*(13.83-27.07)	51.11*(43.84-59.24)
60-119	3.52*(1.42-7.26)	11.64*(8.2-16.05)
120+	4.54*(2.48-7.62)	7.9*(5.56-10.88)
**Era of diagnosis, year**		
1992-2001	14.6*(11.56-18.19)	30.71*(27.18-34.57)
2002-2016	14.87*(9.8-21.64)	40.14*(33.22-48.07)
**Ann Arbor stage**		
I/II	9.18*(6.82-12.11)	22.93*(19.01-27.42)
III/IV	33.06*(24.77-43.25)	41.47*(36.5-46.94)
**lymphoma subtype**		
HL	3.63*(2.07-5.89)	12.88*(10.17-16.1)
DLBCL	31.53*(24.43-40.04)	72.12*(63.36-81.76)
BL	204.38*(116.82-331.9)	116.59*(84.37-157.04)
FL	7.85*(1.62-22.93)	6.01*(2.42-12.39)
MZL	0(0-79.92)	31.36*(11.51-68.26)
MCL	0(0-531.98)	22.5(0.57-125.35)
CLL/SLL	0(0-77.85)	11.92*(3.87-27.82)
PTCL	29.88*(8.14-76.5)	28.58*(13.07-54.26)

*P<0.05

Abbreviations: AYA, adolescent and young adult; SMR, standardized mortality ratio; CI, confidence interval; HL, Hodgkin lymphoma; DLBCL, diffuse large B-cell lymphoma; MCL, mantle cell lymphoma; BL, Burkitt's lymphoma; MZL, marginal zone lymphoma; CLL/SLL, chronic lymphocytic leukemia/small lymphocytic lymphoma; PTCL, peripheral T-cell lymphoma; FL, follicular lymphoma.
