# Supplementary material for: Role of Radiation Therapy in Mortality among Adolescents and Young Adults with Lymphoma: Differences According to Cause of Death
Source: Cancers (Basel). 2022 Oct 16;14(20):5067. doi: 10.3390/cancers14205067 (PMC9599966; doi:10.3390/cancers14205067)
Supplement: Supplementary file 1 [file cancers-14-05067-s001.zip › Table S4.pdf]

Table S4. Standardized mortality ratios of CVD-related mortality among AYA patients according to baseline characteristics.

Characteristic	Radiation	No Radiation
	SMR(95% CI)	SMR(95% CI)
**Overall**	2.42*(1.82-3.16)	2.8*(2.28-3.42)
**Age, y**		
15-24	5.63*(3-9.63)	5.83*(3.39-9.33)
25-39	2.05*(1.47-2.78)	2.53*(2.01-3.14)
**Sex**		
Male	1.99*(1.35-2.82)	2.59*(2.01-3.29)
Female	3.42*(2.17-5.13)	3.41*(2.32-4.84)
**Race**		
White	2.36*(1.7-3.18)	2.61*(2.03-3.31)
Black	2.26(0.98-4.46)	2.82*(1.77-4.27)
Other	4.19*(1.14-10.73)	7.13*(3.08-14.05)
**Latency periods, m**		
0-11	2.64(0.07-14.71)	8.06*(2.96-17.54)
12-59	0.57(0.07-2.07)	3.26*(1.99-5.03)
60-119	2.48*(1.36-4.17)	2.64*(1.67-3.96)
120+	2.89*(2.03-3.98)	2.53*(1.87-3.35)
**Era of diagnosis, year**		
1992-2001	2.87*(2.11-3.82)	2.68*(2.08-3.4)
2002-2016	1.18(0.47-2.42)	3.12*(2.1-4.45)
**Ann Arbor stage**		
I/II	1.9*(1.3-2.69)	2.32*(1.62-3.21)
III/IV	3.63*(2.15-5.73)	3.33*(2.53-4.29)
**lymphoma subtype**		
HL	2.69*(1.89-3.73)	3.18*(2.38-4.15)
DLBCL	1.77(0.91-3.09)	2.89*(1.95-4.13)
BL	0(0-13.85)	5.07*(1.86-11.04)
FL	1.68(0.2-6.08)	0.77(0.16-2.26)
MZL	13.67*(1.66-49.39)	3.5(0.42-12.63)
MCL	0(0-131.11)	0(0-29.96)
CLL/SLL	18.79*(2.28-67.88)	3.32(0.9-8.5)
PTCL	0(0-8.78)	0(0-3.93)

*P<0.05

Abbreviations: AYA, adolescent and young adult; CVD, cardiovascular disease; SMR, standardized mortality ratio; CI, confidence interval; HL, Hodgkin lymphoma; DLBCL, diffuse large B-cell lymphoma; MCL, mantle cell lymphoma; BL, Burkitt's lymphoma; MZL, marginal zone lymphoma; CLL/SLL, chronic lymphocytic leukemia/small lymphocytic lymphoma; FL, follicular lymphoma.
