# Supplementary figures and images for: Role of Radiation Therapy in Mortality among Adolescents and Young Adults with Lymphoma: Differences According to Cause of Death
Source: Cancers (Basel). 2022 Oct 16;14(20):5067. doi: 10.3390/cancers14205067 (PMC9599966; doi:10.3390/cancers14205067)

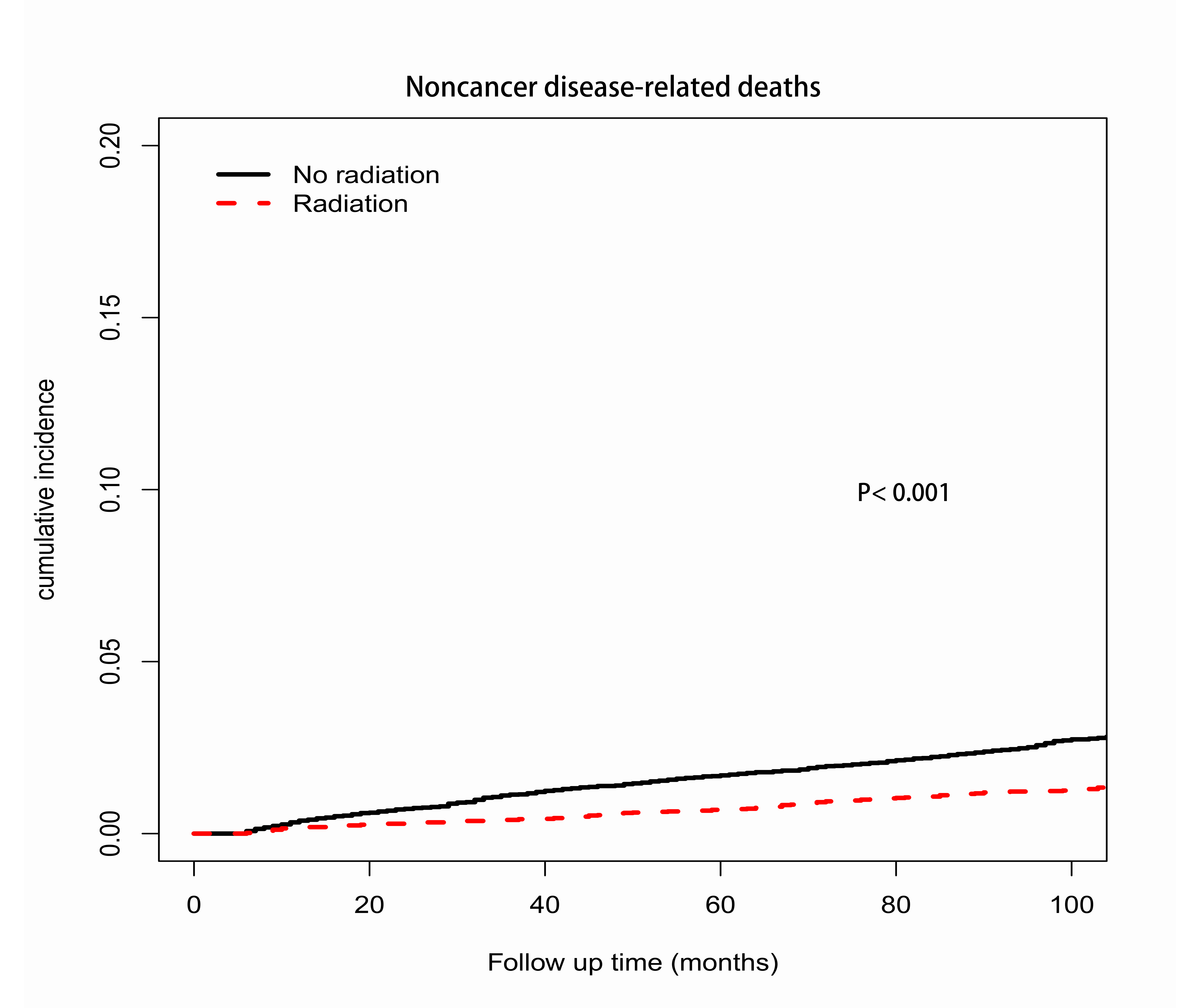

Supplement: Supplementary file 1 [file cancers-14-05067-s001.zip › Figure S1.tif]
